# Predictive role of pre-thrombolytic hs-CRP on the safety and efficacy of intravenous thrombolysis in acute ischemic stroke

**DOI:** 10.1186/s12883-023-03291-7

**Published:** 2023-06-23

**Authors:** Xu-Dong Cheng, Duo-Zi Wang, Qi Zhang, Jian-Hong Wang, Bing-Hu Li, Xin Zhang, Jing Zhang, Sen Zhou, Li-Jun Jia, Li-Rong Wang, Neng-Wei Yu

**Affiliations:** 1 School of Clinical Medicine, Southwest Medical University, Luzhou, 646000 China; 2 Department of Neurology, Sichuan Provincial People’s Hospital, University of Electronic Science and Technology of China, Chengdu, 610072 China

**Keywords:** Acute ischaemic stroke, Intravenous thrombolysis, High sensitivity C-reactive protein, Recombinant plasminogen activator, Urokinase

## Abstract

**Purpose:**

To investigate the predictive role of pre-thrombolytic high sensitivity C-reactive protein (hs-CRP) on the safety and efficacy of intravenous thrombolysis in patients with acute ischemic stroke (AIS).

**Methods:**

Patients with AIS who underwent intravenous thrombolysis with recombinant plasminogen activator (rtPA) or urokinase without endovascular therapy from June 2019 to June 2022 were retrospectively analysed. All patients were grouped into two groups (high or low hs-CRP group) according to the median value of hs-CRP before intravenous thrombolysis. The baseline NIHSS, NIHSS changes before and after thrombolysis (ΔNIHSS), the rate of good thrombolysis response (NIHSS decreased ≥ 2 points from baseline), the rate of any intracranial hemorrhage, age, sex, hypertension, diabetes, uric acid and platelet count were compared between the two groups. Logistic regression analysis was performed to identify possible prognostic factors for a good thrombolysis response.

**Results:**

A total of 212 patients were included in the analysis, with a mean age of 66.3 ± 12.5 years. In total, 145 patients received rtPA, and 67 patients received urokinase. Patients were divided into a high hs-CRP group (> 1.60 mg/L) and a low hs-CRP group (≤ 1.60 mg/L) according to the median hs-CRP level (1.60 mg/L). The ΔNIHSS of the high hs-CRP group was significantly smaller than that of the low hs-CRP group (0 [-1 ~ 0] vs. -1 [-2 ~ 0], P < 0.05). The good rate of thrombolysis response in the high hs-CRP group was significantly lower than that in the low hs-CRP group (21.9% vs. 36.5%, P < 0.05). Similar results were shown in the rtPA subgroup between the high and low hs-CRP groups but not in the urokinase subgroup. Logistic regression analysis showed that hs-CRP > 1.60 mg/L was negatively correlated with a good thrombolysis response rate (OR = 0.496, 95% CI = 0.266–0.927, P = 0.028).

**Conclusion:**

hs-CRP > 1.6 mg/L may serve as a poor prognosis predictive factor for patients with AIS receiving intravenous thrombolysis. However, due to the small sample size of this study, further studies are needed to verify our results.

## Introduction

Acute ischemic stroke (AIS) is the leading cause of disability and death in China due to its rapid onset, high mortality, recurrence rate and disability rate [1]. Intravenous thrombolysis is one of the most effective treatments for AIS and can quickly restore the blood supply to the brain, shorten the ischemic damage time, and restore neurological deficits [2, 3]. However, clinical studies have shown that only 20–30% of patients achieve complete recanalization 2 h after thrombolysis, up to 60% of patients only achieve partial recanalization, and 34% of them may be occluded again [4]. Therefore, it is urgent to explore factors that can predict the safety and efficacy of intravenous thrombolysis.

Mechanistically, the pathogenesis of AIS is complex and may be related to lipid regulation, blood anticoagulation and the fibrinolytic system [5]. Recent studies have shown that inflammation is involved in the development and progression of AIS [6, 7]. In terms of inflammation, studies have shown that increased white blood cell count is significantly associated with poor prognosis [8]. In addition, high sensitivity C-reactive protein (hs-CRP) has been reported to be associated with the severity and prognosis of AIS. Moreover, with the decrease in hs-CRP concentration, the proportion of neurological improvement seemed to be increased [9]. However, the significance of hs-CRP level before intravenous thrombolysis in the prognosis of thrombolysis has not been reported. Therefore, this study intended to investigate the predictive role of pre-thrombolytic hs-CRP on the safety and efficacy of intravenous thrombolysis in patients with AIS.

## Methods

### Patients

Patients with AIS who underwent intravenous thrombolysis in the Department of Neurology, Sichuan Academy of Medical Sciences • Sichuan Provincial People’s Hospital from June 2019 to June 2022 were retrospectively analysed. The inclusion/exclusion criteria of the study were as follows: (1) patients met the diagnostic criteria for AIS confirmed by head CT or MRI and complied with guidelines for early management of patients with acute ischemic stroke [10, 11]; (2) the time from onset to intravenous thrombolysis was less than 6 h; (3) National Institute of Health Stroke Scale (NIHSS) scores were obtained before and 24 h after thrombolysis; (4) patients with other stroke etiologies (including infectious, immune, genetic, vascular, and drug use caused stroke) according the TOAST classification [12] were excluded; (5) patients with neurological impairment due to intracranial tumours or craniocerebral trauma were excluded; (6) patients received endovascular treatment were excluded; and (7) studies with incomplete data (no pre-thrombolysis or post-thrombolysis NIHSS score; hs-CRP were not examined before thrombolysis) were excluded. This study was approved by the Ethical Review Board.

### Data collection

Demographic data (gender and age) and cerebrovascular risk factors (hypertension and diabetes) were collected. NIHSS score, serum hs-CRP, uric acid, platelet count and other data were collected before intravenous thrombolysis. The incidence of spontaneous intracranial haemorrhage 24 h after thrombolysis was collected, which was classified according to the European Cooperative Acute Stroke Study III (ECASS III) [13]. NIHSS scores were collected 24 h after thrombolysis. Diabetes mellitus was defined as treated or presently diagnosed according to the 1999 World Health Organization criteria (fasting blood glucose of ≥ 7.0 mmol/L, or 2-hour oral glucose tolerance test glucose of ≥ 11.1 mmol/L) or a history of hyperglycemia managed by insulin, oral hypoglycemic agents, or diet. We defined hypertension as persistent systolic blood pressure ≥ 140 mmHg or diastolic blood pressure ≥ 90 mmHg, a clear history of hypertension on medication, or both.

### Outcomes

The efficacy outcomes included ΔNIHSS score (NIHSS score at 24 h after thrombolysis therapy - NIHSS score before thrombolysis therapy) and good thrombolysis response rate (ΔNIHSS≤-2). Secondary safety outcomes included any intracranial hemorrhage within 24 h.

**1.4 Statistical Methods** SPSS 24.0 statistical software was used for data analysis. Data with a normal distribution are expressed as the mean ± standard deviation (X ± S), and Student’s t test was used. Data with a nonnormal distribution are expressed as the median and interquartile range [M (Q25, Q75)], and the Mann‒Whitney U test was used. The χ2 test or Fisher’s exact test was used to compare categorical variables. Logistic regression analysis was performed to identify predictors for a good thrombolysis response rate. P < 0.05 was considered statistically significant.

## Results

### General data of patients

A total of 212 patients were enrolled in this study, with an average age of 66.3 ± 12.5 years, including 134 male patients and 78 female patients. A total of 142 patients had hypertension, and 71 patients had diabetes. Sixty-seven patients received urokinase, and 145 received rtPA. According to the TOAST classification [12], there were 95 patients with large-artery atherosclerosis (LAA), 60 patients with small-artery occlusion (SAO), 45 patients with cardioembolism (CE) and 12 patients with undetermined etiology (SUE). The median (interquartile range) NIHSS score was 4 (2 ~ 8) points at baseline and 3 (2 ~ 6) points after thrombolysis. The median (interquartile range) ΔNIHSS score was 0 (-2 ~ 0) points. The median hs-CRP was 1.60 mg/L. The 25% quantile was 0.72 mg/L, and the 75% quantile was 3.64 mg/L. According to previous studies [14–16] and the median hs-CRP level of our cohort, the cut-off point of hs-CRP was preset as 1.6 mg/L, and all the patients were divided into a high CRP group (hs-CRP > 1.60 mg/L) and a low CRP group (hs-CRP ≤ 1.60 mg/L).

### Comparisons between the high and low hs-CRP groups

As shown in Table 1, there was no significant difference in age, sex, hypertension, diabetes, uric acid, platelet count, baseline NIHSS score, or any intracranial hemorrhage between the high and low hs-CRP groups (P > 0.05). The ΔNIHSS of the high hs-CRP group was significantly smaller than that of the low hs-CRP group (0 [-1 ~ 0] vs. -1 [-2 ~ 0], P < 0.05). The good thrombolysis response rate of the high hs-CRP group was significantly lower than that of the low hs-CRP group (21.9% vs. 36.5%, P < 0.05).

**Table 1:** Comparisons between the high and low hs-CRP groups

	High hs-CRP (n = 105)	Low hs-CRP (n = 107)	P value
Age (years), mean ± SD	67.4 ± 11.86	65.27 ± 13.04	0.231
Male, n (%)	66 (62.8%)	68 (63.5%)	0.917
Hypertension, n (%)	75 (71.4%)	67 (63.8%)	0.173
Diabetes, n (%)	32 (30.4%)	39 (36.4%)	0.357
Uric acid (µmol/L), Median (IQR)	348 (290–411)	363 (298–410)	0.585
Platelet count (10^9/L), Median (IQR)	194 (146–235)	176 (140–208)	0.017
Baseline NIHSS	4 (2 ~ 8)	4 (2 ~ 8)	0.695
ΔNIHSS, Median (IQR)	0 (-1 ~ 0)	-1 (-2 ~ 0)	<0.001
Good thrombolysis response rate, n (%)	23 (21.9%)	39 (36.5%)	0.02
Any intracranial hemorrhage, n (%)	13 (12.4%)	12 (11.2%)	0.792
NIHSS, National Institutes of Health Stroke Scale; IQR, interquartile range; SD, standard deviation			

### Comparisons between the high and low hs-CRP groups in patients treated with rtPA

As shown in Table 2, in patients treated with rtPA, there was no significant difference in age, sex, hypertension, diabetes, uric acid, platelet count, baseline NIHSS score or any intracranial hemorrhage between the high and low hs-CRP groups (P > 0.05). The ΔNIHSS of the high hs-CRP group was significantly smaller than that of the low hs-CRP group (0 [-1.75 ~ 0] vs. -1 [-2 ~ 0], P < 0.05). The good thrombolysis response rate of the high hs-CRP group was significantly lower than that of the low hs-CRP group (25% vs. 42.9%, P < 0.05).

**Table 2:** Comparisons between the high and low hs-CRP groups in patients with rtPA

	High hs-CRP (n = 68)	Low hs-CRP (n = 77)	P value
Age (years), mean ± SD	68.74 ± 13.16	65.71 ± 13.04	0.168
Male, n (%)	42 (61.8%)	50 (64.9%)	0.692
Hypertension, n (%)	50 (70.6%)	46 (59.7%)	0.172
Diabetes, n (%)	20 (29.4%)	25 (32.5%)	0.691
Uric acid (µmol/L), Median (IQR)	347 (286–397)	365 (301–408)	0.247
Platelet count (10^9/L), Median (IQR)	194 (144–236)	173 (129–205)	0.363
Baseline NIHSS	5 (2 ~ 8)	4 (2 ~ 9)	0.476
ΔNIHSS, Median (IQR)	0 (-1.75 ~ 0)	-1 (-2 ~ 0)	0.002
Good thrombolysis response rate, n (%)	17 (25%)	33 (42.9%)	0.024
Any intracranial hemorrhage, n (%)	9 (13.2%)	9 (11.7%)	0.778

NIHSS, National Institutes of Health Stroke Scale; IQR, interquartile range; SD, standard deviation.

### Comparisons between the high and low hs-CRP groups in patients treated with urokinase

As shown in Table 3, in patients treated with urokinase subgroup, there was no significant difference between the high and low hs-CRP groups in age, gender, hypertension, diabetes, uric acid, platelet count, baseline NIHSS score, ΔNIHSS, good thrombolysis response rate, and any intracranial hemorrhage (P > 0.05).

**Table 3:** Comparisons between the high and low hs-CRP in patients treated with urokinase

	High hs-CRP (n = 37)	Low hs-CRP (n = 30)	P value
Age (years), mean ± SD	64.97 ± 8.66	64.13 ± 13.22	0.756
Male, n (%)	24 (64.8%)	18 (60%)	0.682
Hypertension, n (%)	27 (73%)	21 (70%)	0.788
Diabetes, n (%)	12 (32.4%)	14 (46.7%)	0.234
Uric acid (µmol/L), Median (IQR)	365 (317–426)	357 (281–425)	0.623
Platelet count (10^9/L), Median (IQR)	191 (158–232)	186(161–226)	0.641
Baseline NIHSS	4 (2 ~ 5)	4 (2 ~ 5.25)	0.799
ΔNIHSS, Median (IQR)	0 (-1 ~ 0)	-0.5 (-1 ~ 0)	0.223
Good thrombolysis response rate, n (%)	6 (16.2%)	6 (20%)	0.688
Any intracranial hemorrhage, n (%)	4 (10.8%)	3 (10%)	0.914

NIHSS, National Institutes of Health Stroke Scale; IQR, interquartile range; SD, standard deviation.

### Comparisons between the high and low hs-CRP groups in patients with LAA subgroup

A total of 105 patients with LAA were included in this study, including 50 patients in the high hs-CRP group and 45 patients in the low hs-CRP group. As shown in Table 4, there was no significant difference in age, sex, hypertension, diabetes, uric acid, platelet count, baseline NIHSS score, good thrombolysis response or any intracranial hemorrhage between the high and low hs-CRP groups (P > 0.05). The ΔNIHSS of the high hs-CRP group was significantly smaller than that of the low hs-CRP group (0 [-1.25 ~ 0] vs. -1 [-2 ~ 0], P < 0.05). The platelet count of the low hs-CRP group was significantly smaller than that of the high hs-CRP group (176 [147-206.5] vs. 205 [159.25–244], P < 0.05).

**Table 4:** Comparisons between the high and low hs-CRP groups in patients with LAA subgroup

	High hs-CRP (n = 50)	Low hs-CRP (n = 45)	P value
Age (years), mean ± SD	67.94 ± 10.61	66.07 ± 14.01	0.464
Male, n (%)	40 (80%)	30 (66.7%)	0.501
Hypertension, n (%)	40 (80%)	29 (70%)	0.09
Diabetes, n (%)	21 (42%)	14 (31.1%)	0.272
Uric acid (µmol/L), Median (IQR)	329 (264.25–416)	354 (276.5–409)	0.743
Platelet count (10^9/L), Median (IQR)	205 (159.25–244)	176 (147-206.5)	0.026
Baseline NIHSS	4 (2.75 ~ 7.25)	5 (2 ~ 9.5)	0.422
ΔNIHSS, Median (IQR)	0 (-1.25 ~ 0)	-1 (-2 ~ 0)	0.007
Good thrombolysis response rate, n (%)	12 (24%)	18 (40%)	0.094
Any intracranial hemorrhage, n (%)	6 (12%)	9 (20%)	0.286

NIHSS, National Institutes of Health Stroke Scale; IQR, interquartile range; SD, standard deviation

### Comparisons between the high and low hs-CRP groups in the SAO subgroup

A total of 60 patients with SAO were included in this study, including 30 patients in the high hs-CRP group and 30 patients in the low hs-CRP group. As shown in Table 5, there was no significant difference between the high and low hs-CRP groups in age, sex, hypertension, diabetes, uric acid, platelet count, baseline NIHSS score, ΔNIHSS, good thrombolysis response rate, or any intracranial hemorrhage (P > 0.05).

**Table 5:** Comparisons between the high and low hs-CRP groups in SAO subgroup

	High hs-CRP (n = 30)	Low hs-CRP (n = 30)	P value
Age (years), mean ± SD	64.4 ± 11.46	62.97 ± 10.41	0.614
Male, n (%)	22 (73.3%)	21 (70%)	0.774
Hypertension, n (%)	20 (66.7%)	18 (60%)	0.592
Diabetes, n (%)	5 (16.7%)	10 (33.3%)	0.136
Uric acid (µmol/L), mean ± SD	344 ± 79.82	363.77 ± 72.75	0.743
Platelet count (10^9/L), mean ± SD	191.73 ± 50.51	187.87 ± 53.97	0.704
Baseline NIHSS	3 (2 ~ 6.25)	3 (1.75 ~ 5)	0.255
ΔNIHSS, Median (IQR)	-0.5 (-1.25 ~ 0)	-1 (-2 ~ 0)	0.73
Good thrombolysis response rate, n (%)	7 (30.4%)	8 (26.7%)	0.766
Any intracranial hemorrhage, n (%)	2 (6%)	2 (6%)	1.0

NIHSS, National Institutes of Health Stroke Scale; IQR, interquartile range; SD, standard deviation.

### Comparisons between the high and low hs-CRP groups in the CE subgroup

A total of 45 patients with CE were included in this study, including 21 patients in the high hs-CRP group and 24 patients in the low hs-CRP group. As shown in Table 6, there was no significant difference in age, sex, hypertension, diabetes, uric acid, platelet count, baseline NIHSS score, or any intracranial hemorrhage between the high and low hs-CRP groups (P > 0.05). The ΔNIHSS of the high hs-CRP group was significantly smaller than that of the low hs-CRP group (0 [0 ~ 0] vs. -1 [-2.75 ~ 0], P < 0.05). The good thrombolysis response rate of the high hs-CRP group was significantly lower than that of the low hs-CRP group (9.5% vs. 33.3%, P < 0.05).

**Table 6:** Comparisons between the high and low hs-CRP groups in CE subgroup

	High hs-CRP (n = 21)	Low hs-CRP (n = 24)	P value
Age (years), mean ± SD	70.81 ± 14.82	70.63 ± 11.77	0.964
Male, n (%)	11 (52.4%)	14 (58.3%)	0.688
Hypertension, n (%)	14 (66.7%)	16 (66.7%)	1.0
Diabetes, n (%)	3 (14.28%)	12 (50%)	0.01
Uric acid (µmol/L), mean ± SD	379.81 ± 84.43	393.63 ± 88.51	0.596
Platelet count (10^9/L), mean ± SD	192.81 ± 73.99	157 ± 35.85	0.053
Baseline NIHSS	7 (3 ~ 17)	5 (2.25 ~ 8.75)	0.343
ΔNIHSS, Median (IQR)	0 (0 ~ 0)	-1 (-2.75 ~ 0)	0.005
Good thrombolysis response rate, n (%)	2 (9.5%)	8 (33.3%)	0.05
Any intracranial hemorrhage, n (%)	4 (19.04%)	0 (0%)	0.025

NIHSS, National Institutes of Health Stroke Scale; IQR, interquartile range; SD, standard deviation

### Comparisons between the high and low hs-CRP groups in the SUE subgroup

A total of 12 patients with SUE were included in this study, including 4 patients in the high hs-CRP group and 8 patients in the low hs-CRP group. As shown in Table 7, there was no significant difference between the high and low hs-CRP groups in age, sex, hypertension, diabetes, uric acid, platelet count, baseline NIHSS score, ΔNIHSS, good thrombolysis response rate, or any intracranial hemorrhage (P > 0.05).

**Table 7:** Comparisons between the high and low hs-CRP groups in SUE subgroup

	High hs-CRP (n = 4)	Low hs-CRP (n = 8)	P value
Age (years), mean ± SD	65.5 ± 11.12	53.38 ± 11.70	0.117
Male, n (%)	3 (75%)	3 (37.5%)	0.221
Hypertension, n (%)	1 (25%)	4 (50%)	0.408
Diabetes, n (%)	3 (75%)	3 (37.5%)	0.221
Uric acid (µmol/L), mean ± SD	431 ± 86.35	358.25 ± 91.73	0.217
Platelet count (10^9/L), mean ± SD	165.5 ± 71.94	197.88 ± 85.26	0.531
Baseline NIHSS	3.25 ± 1.50	4.86 ± 3.36	0.907
ΔNIHSS, Median (IQR)	-1.75 ± 2.06	-1.75 ± 1.75	1.0
Good thrombolysis response rate, n (%)	2 (50%)	5 (62.5%)	0.679
Any intracranial hemorrhage, n (%)	1 (25%)	1 (12.5%)	0.584

NIHSS, National Institutes of Health Stroke Scale; IQR, interquartile range; SD, standard deviation

### Multivariate logistic regression analysis of predictors for good thrombolysis response rate

A good thrombolysis response rate was considered the dependent variable. Age, sex, hypertension, diabetes, uric acid, etiological classification of acute ischemic stroke, platelet count, and hs-CRP were considered independent variables. As shown in Table 8, hs-CRP was an independent risk factor for a good thrombolysis response (OR = 0.496, 95% CI = 0.266–0.927, P = 0.028).

**Table 8:** Multivariate logistic regression analysis of predictors for good thrombolysis response rate

Factors	B	SE	Waldχ2	P value	OR	95% CI
Age	0.025	0.014	3.012	0.83	1.025	0.997–1.055
Male	0.634	0.372	2.897	0.89	1.885	0.908–3.911
Hypertension	0.610	0.361	2.858	0.91	1.841	0.907–3.734
Diabetes	-0.129	0.352	0.135	0.741	0.879	0.441–1.752
Uric acid	-0.001	0.002	0.243	0.622	0.999	0.995–1.003
Platelet	0.000	0.002	0.057	0.811	1.000	0.996–1.005
hs-CRP > 1.6 mg/L	-0.716	0.326	4.84	0.028	0.489	0.258–0.925
TOAST classification: LAA			6.831	3	0.077	
TOAST classification: SUE	1.193	0.676	3.116	0.078	3.297	0.877–12.401
TOAST classification: SAO	-0.367	0.387	0.901	0.342	0.693	0.325–1.478
TOAST classification: CE	-0.593	0.453	1.71	0.191	0.553	0.227–1.344

OR, Odd Ratio; CI, Confidence Interval; LAA, large-artery atherosclerosis; SUE, undetermined aetiology; SAO, small-artery occlusion; CE, cardioembolism.

## Discussion

Currently, intravenous thrombolysis is one of the most effective therapies for AIS and can improve or even completely restore obstructed blood vessels. However, clinical practice has found that the recanalization rate of intravenous thrombolysis with alteplase is low, resulting in a poor prognosis [17, 18]. Therefore, it is urgent to find the influencing factors or predictors of intravenous thrombolysis. The results of this study showed that the median ΔNIHSS in the low hs-CRP group (≤ 1.60 mg/L) was significantly larger than that in the high hs-CRP group (> 1.60 mg/L), and the good thrombolysis response rate was also higher than that in the high hs-CRP group. The same result was found in the rtPA subgroup but not in the urokinase subgroup. The results of logistic regression analysis showed that hs-CRP > 1.60 mg/L was significantly correlated with poor prognosis 24 h after thrombolysis.

hs-CRP is a factor synthesized and secreted by the liver and is a nonspecific marker reflecting the acute phase of the systemic inflammatory response [19]. Preclinical studies have found that hs-CRP is involved in atherosclerotic plaque formation and thrombosis. During the formation of atherosclerotic plaques, CRP and other inflammatory factors are deposited in the artery wall, which further activate complement, produce a large number of inflammatory mediators, release oxygen free radicals, and cause intimal injury of blood vessels [20]. The role of hs-CRP in atherosclerotic injury is far beyond the role of inflammatory mediators, which can not only induce the secretion of inflammatory factors but also induce the rupture of unstable atherosclerotic plaques, resulting in embolism and thrombosis [21]. In terms of stroke, hs-CRP has been shown to play an important role in predicting prognosis and risk factors for stroke, including long- and short-term mortality risk, early diagnosis, etiological subtype prediction, and prognosis [22]. Moreover, high hs-CRP levels in patients with AIS who underwent intravenous thrombolysis were associated with adverse outcomes and mortality at 3 months [23]. Some studies focusing on hs-CRP 24 h after thrombolysis showed that a high level of hs-CRP was associated with adverse functional outcomes (modified Rankin score ≥ 3) after 3 months [24]. The results from our study showed that the rate of good thrombolysis response in patients with high hs-CRP (> 1.6 mg/L) was significantly lower than that in patients with low hs-CRP. This suggests that as an indicator of inflammation, hs-CRP can reflect the level of inflammation; patients with high levels of inflammation have a poor prognosis after thrombolysis. As a biomolecule, hs-CRP may have a potential effect on thrombolytic agents, which needs further study and exploration.

For ischemic stroke, aetiological subtypes included large-artery atherosclerosis (LAA), small-artery occlusion (SAO), cardioembolism (CE) and undetermined etiology (SUE). Liu et al. [25] found that hs-CRP was significantly different among different aetiological subtypes and was highest in the CE subtype. Our study also made comparisons between the different etiological subtypes. In the CE subgroup, ΔNIHSS and the rate of good thrombolytic response were significantly different between the high and low hs-CRP groups. In the LAA subgroup, although ΔNIHSS was significantly different between the high and low hs-CRP groups; the rate of good thrombolytic response was not significantly different between the high and low hs-CRP groups. Our study suggests that hs-CRP > 1.60 mg/L was significantly correlated with the prognosis 24 h after thrombolysis in patients with AIS, especially the CE subtype.

According to the Guidelines for the Early Management of Patients With Acute Ischemic Stroke [10], rtPA has been widely confirmed as an intravenous thrombolytic agent that can improve the functional prognosis of patients with AIS. However, according to the Chinese Stroke guidelines [11], both rtPA and urokinase have been approved for patients with AIS. Urokinase and rtPA share similar mechanisms of action. The former is a nonselective thrombolytic drug that can inhibit blood coagulation by acting on the endogenous fibrinolytic system and vascular ADP enzyme coagulation system. The latter is a specific fibrinogen activator that can selectively bind fibrin to promote its transformation into plasmin to achieve thrombolysis [26]. Our subgroup analysis showed a significant difference between the high and low hs-CRP patients in the rtPA subgroup in terms of good thrombolysis response rate but not in the urokinase subgroup. We hypothesized that hs-CRP might have different effects on the two above drugs. Previous studies have shown that CRP can induce the expression of plasminogen activator inhibitor type 1 (PAI-1) [27], which is a member of the serine protease inhibitor (SERPIN) superfamily and inhibits the protease activities of plasminogen activators (PAs) by forming complexes with PAs, thereby regulating fibrinolysis [28]. Therefore, we hypothesized that high levels of hs-CRP might affect the thrombolytic effect of rtPA but not urokinase. Therefore, should urokinase be given priority in patients with high hs-CRP?

With the publication of several RCTs focusing on anti-inflammatory therapy in the cardiovascular field in recent years [29, 30], residual inflammation risk has attracted increasing interest in the field of stroke prevention and treatment. Our findings raise the question: should patients with pre-thrombolysis hs-CRP greater than 1.6 mg/L be more aggressively treated with anti-inflammatory therapy?

Although intravenous thrombolysis therapy is one of the most effective treatments to improve or even completely restore blood flow in obstructed vessels, intravenous thrombolysis therapy can increase the blood‒brain barrier permeability and the risk of hemorrhagic transformation (HT) in patients with AIS. Clinical characteristics, such as severity of stroke, advanced age, hypertension, atrial fibrillation, diabetes, renal insufficiency, congestive heart failure, ischemic heart disease, use of antiplatelet drugs, and cerebral microbleeds, are associated with an increased risk of bleeding after thrombolysis [31, 32]. Mechanistically, the pathogenesis of HT has not been fully elucidated, but inflammation and oxidative stress have been reported to be the major contributors [33, 34]. However, our study found that hs-CRP was not associated with bleeding transformation after thrombolysis.

Several limitations should be acknowledged. First, this study only analysed the neurological recovery of patients 24 h after thrombolysis. Long-term prognosis has not been analysed, such as mRS, which is widely used to assess stroke prognosis. Its analysis may provide more valuable information on the safety and efficacy of intravenous thrombolysis in acute ischaemic stroke. Second, the sample size was relatively small, which may lead to bias in the statistical results. Third, this study was a retrospective analysis, which may have some influence on the power of the results.

In conclusion, hs-CRP > 1.6 mg/L may serve as a poor prognosis predictor for patients with AIS receiving intravenous thrombolysis. However, due to the small sample size of this study, further studies are needed to verify our results.

## Data Availability

The datasets in this study are available from the corresponding author on reasonable request.
